# Supplementary material for: Dolutegravir use over 48 weeks is not associated with worsening insulin resistance and pancreatic beta cell function in a cohort of HIV-infected Ugandan adults
Source: AIDS Res Ther. 2023 Sep 9;20:65. doi: 10.1186/s12981-023-00564-6 (PMC10492310; doi:10.1186/s12981-023-00564-6)
Supplement: Supplementary file 1 — Supplementary Material 1 [file 12981_2023_564_MOESM1_ESM.docx]

**Dolutegravir use over 48 weeks is not associated with worsening insulin resistance and pancreatic beta cell function in a cohort of HIV-infected Ugandan adults**

Frank Mulindwa^1,2^, Barbara Castelnuovo^1^, Nele Brusselaers ^2,3,^ Martin Nabwana^4^, Robert Bollinger^5^,Allan Buzibye^1^, , [Eva Laker Agnes Odongpiny](https://onlinelibrary.wiley.com/action/doSearch?ContribAuthorRaw=Odongpiny%2C+Eva+Laker+Agnes)^1^, Ronald Kiguba^6^, and Jean-Marc Schwarz^7,8^

**Affiliations:**

1. *Capacity Building Program, Makerere University Infectious Diseases Institute, Kampala, Uganda.*
2. *Global Health Institute, Antwerp University, Antwerp, Belgium*
3. *Centre for Translational Microbiome Research, Department of Microbiology, Tumour and Cell Biology, Karolinska University, Stockholm, Sweden.*
4. *Makerere University - Johns Hopkins University Research Collaboration, Kampala, Uganda.*
5. *School of Medicine, Johns Hopkins University, Baltimore, USA*
6. *Department of Pharmacology and Therapeutics, College of Health Sciences Makerere University, Kampala, Uganda*
7. *School of Medicine, University of California San Francisco, San Francisco, USA*
8. *Department of Basic Sciences, Touro University California College of Osteopathic Medicine, Vallejo, California, USA.*

**Corresponding author:**

Dr. Frank Mulindwa

Makerere University Infectious Diseases Institute, Uganda.

Global Health Institute, Antwerp University, Antwerp, Belgium

Mailto: [fmulindwa@idi.co.ug](mailto:fmulindwa@idi.co.ug), [mulindwafrank93@gmail.com](mailto:mulindwafrank93@gmail.com)

**Table S1. Factors associated with changes in Changes in pancreatic beta cell function and insulin resistance over 48 weeks**

|  | **Pancreatic beta cell function** | | | | **Insulin Resistance** | | | |
| --- | --- | --- | --- | --- | --- | --- | --- | --- |
| **Characteristic** | **Crude change  (95% CI)** | **P-value** | **Adjusted change  (95% CI)** | **P-value** | **Crude change (95% CI)** | **P-value** | **Adjusted change (95% CI)** | **P-value** |
| Age | 0.68 (-2.05, 3.41) | 0.621 | 1.82 (-1.72, 5.36) | 0.305 | -0.01 (-0.091, 0.08) | 0.900 | 0.03 (-0.08, 0.14) | 0.616 |
| Baseline CD4 | -0.03 (-0.12, 0.06) | 0.485 | -0.03 (-0.13, 0.06) | 0.493 | -0.001 (-0.003, 0.002) | 0.561 | -0.001 (-0.004, 0.002) | 0.499 |
| Waist circumference | -1.40 (-4.04, 1.24) | 0.291 | -4.52 (-8.47, -0.58) | 0.026 | -0.07 (-0.15, 0.008) | 0.078 | -0.15 (-0.28, -0.02) | 0.020 |
| Sex |  |  |  |  |  |  |  |  |
| Female | Ref |  |  |  |  |  |  |  |
| Male | 13.09 (-27.53, 53.72) | 0.521 | 4.77 (-47.74, 57.28) | 0.855 | 0.25 (-0.97, 1.47) | 0.682 | 0.16 (-1.46, 1.78) | 0.847 |
| Body Mass Index (BMI) |  |  |  |  |  |  |  |  |
| Underweight (<18.5) | Ref |  |  |  |  |  |  |  |
| Normal (18.5-24.9) | 7.42 (-54.53, 69.36) | 0.811 | 56.43 (-20.14, 133.0) | 0.144 | 0.34 (-1.63, 2.31) | 0.731 | 1.76 (-0.69, 4.21) | 0.155 |
| Overweight (25.0-29.9) | 2.68 (-75.26, 80.63) | 0.945 | 89.59 (-20.56, 199.73) | 0.108 | -0.21 (-2.44, 2.03) | 0.855 | 2.67 (-0.73, 6.08) | 0.121 |
| Obese (≥30) | 76.74 (-84.27, 237.74) | 0.343 | 207.59 (5.40, 409.79) | 0.044 | 1.36 (-3.80, 6.52) | 0.601 | 5.70 (-0.78, 12.19) | 0.083 |
| Physical activity |  |  |  |  |  |  |  |  |
| GPAQ<600 MET minutes | Ref |  |  |  |  |  |  |  |
| GPAQ≥600 MET minutes | 43.81 (-19.5, 107.14) | 0.171 | 44.62 (-23.93, 113.16) | 0.196 | 1.20 (-0.58, 2.99) | 0.181 | 1.21 (-0.72, 3.15) | 0.213 |
